# Analysis of Zona-Associated Sperm as a Potential Source of Exogenous *Y-DNA* Interference in Sex-Typing of In Vitro-Fertilized Ovine Embryos

**DOI:** 10.3390/ani16182888

**Published:** 2026-09-14

**Authors:** Qiangqiang Ma, Ying Chen, Hong Dong, Liqin Wang, Xuefeng Fu, Gulimire Abudureyimu, Jiaxin Zhang, Wei Zhang, Jinbo Tian, Yangsheng Wu, Jiapeng Lin

**Affiliations:** 1 College of Animal Science, Xinjiang Agricultural University, Urumqi 830052, China; xjwh_mq@163.com (Q.M.); zjxin0920@163.com (J.Z.);; 2 Key Laboratory of Genetic Breeding and Reproduction of Herbivorous Livestock, Ministry of Agriculture and Rural Affairs/Xinjiang Key Laboratory of Reproductive Regulation and Breeding for Ruminant Livestock/Xinjiang Key Laboratory of Animal Biotechnology, Xinjiang Academy of Animal Science, Urumqi 830011, China

**Keywords:** PCR-based sex classification, AMEL gene, embryo, sperm, zona pellucida

## Abstract

This study aimed to explore whether zona-associated residual sperm may contribute to exogenous *Y-DNAY-DNA* contamination and cause sex bias in PCR-based sex classification of ovine early-stage embryos, based on the amplification of the *AMEL* gene. Primers targeting the conserved regions of the ovine *AMELX* and *AMELY* genes were designed, and a nested PCR system using AMEL and *YWHAZ* genes was established. Group A comprised 17 female and 33 male embryos, corresponding to a female-to-male ratio of 0.52:1, whereas Group B comprised 23 female and 27 male embryos, corresponding to a female-to-male ratio of 0.85:1. DAPI staining revealed sperm-morphology structures or structures containing sperm-derived DNA adjacent to the zona pellucida of ovine early-stage embryos. Sequence alignment of PCR amplicons showed consistency with the *AMELX* and *AMELY* reference genes. In conclusion, these results support our research hypothesis that zona-associated residual sperm may serve as a potential source of *Y-DNAY-DNA* contamination and interfere with PCR-based embryonic sex determination.

## Simple Summary

Sex determination of early mammalian embryos can improve the efficiency of livestock production. In this study, a nested PCR system targeting the *AMEL* gene was established for sex classification of ovine early-stage embryos, and the potential role of zona-associated residual sperm in male-biased genotyping was evaluated. Residual sperm were observed on the zona pellucida surface of zona-intact embryos, and Y-chromosomal DNA signals were detected in partial zona-washing fluids. These findings are consistent with our hypothesis that zona-trapped residual sperm act as a potential source of *Y-DNA* contamination, and with the observed male-biased results in PCRv-based sex genotyping. However, since the PCR-based sex-typing results were not validated by an independent gold-standard method, a causal relationship cannot be established. Zona-associated residual sperm should therefore be considered a potential source of interference rather than the sole cause of the observed bias.

## 1. Introduction

In animal husbandry, early-stage embryo sex-identification technology is of great significance for optimizing reproductive strategies and improving production efficiency. In economically important livestock species such as sheep, embryo sex determination enables the selective production of offspring of a desired sex to meet market demands, for example, for meat or dairy production [1]. Sex determination is generally performed prior to embryo transfer to ensure that only embryos of the desired sex are transferred into recipient females. Currently, molecular-biology-based approaches dominate embryo sex-diagnosis research, with polymerase chain reaction (PCR) amplification being widely favored owing to its simplicity and high reliability [2]. Nested PCR is especially valuable for sex genotyping using single embryonic cells or limited-quantity DNA samples. This high-sensitivity and high-specificity assay adopts two successive rounds of amplification with two pairs of primers, markedly improving detection efficiency and accuracy for trace-input DNA, and effectively overcoming challenges caused by low DNA template quantity and potential PCR inhibitors [3]. Nested-PCR-based sexing has been successfully applied to 2-cell, 4-cell, 8-cell and 16-cell mouse embryos [4].

The amelogenin gene (*AMEL*) is among the widely used target genes for PCR-based sex classification in various mammalian species, including sheep [2], buffalo [4], goats [5], and cattle [6]. *AMEL* exists on both X and Y chromosomes, exhibiting sex-specific sequence polymorphisms, primarily manifested as length variations between *AMELX* (X-chromosome copy) and *AMELY* (Y-chromosome copy). In sheep, for instance, base deletions within the fifth intron of *AMELY* produce a PCR amplicon approximately 100–150 bp shorter than the *AMELX* product [7]. Such length differences allow clear discrimination between female (XX) and male (XY) embryos via agarose gel electrophoresis or melting-curve-based assays, such as high-resolution melting analysis [8]. In goats, female DNA yields a single 280 bp fragment, whereas male DNA generates both 280 bp and 235 bp amplicons [5]. Similarly, *AMEL* PCR in buffalo displays sex-specific banding patterns, with females producing only a 312 bp fragment and males producing 312 bp and 248 bp products [6]. Previous studies have suggested that *AMEL*-based PCR provides high specificity and accuracy (>99%) for ovine embryo sexing [2].

Nevertheless, this technique relies on trace amounts of embryonic DNA and is highly susceptible to exogenous DNA contamination, which may result in erroneous sex assignments [9]. This susceptibility is particularly pronounced in ultra-low-input systems involving single early-stage embryos, where total DNA input is only 0.6–1.2 ng. Even trace amounts of residual Y-chromosome DNA can, therefore, trigger severe false-positive male calls [10].

The zona pellucida (ZP), a non-cellular glycoprotein matrix surrounding mammalian oocytes and early-stage embryos, exhibits multiple essential functions during fertilization, including mediating sperm–oocyte recognition, blocking polyspermy, and protecting pre-implantation embryos [11,12]. During fertilization, numerous spermatozoa accumulate around the oocyte to form a “sperm cloud,” from which only a single sperm successfully penetrates the ZP and achieves fertilization. The remaining sperm bind specifically to ZP3 glycoprotein receptors and may remain firmly attached to the outer ZP surface or become embedded within the matrix gaps [13]. Therefore, the potential for ZP-retained sperm to serve as a source of *Y-DNA* contamination during subsequent molecular detection has been explored in recent years.

Chemical or mechanical ZP removal (e.g., acid Tyrode’s solution or pronase treatment) improves accessibility to embryonic cells, which is critical for assays involving trace-quantity DNA templates [14]. In addition to improving DNA extraction efficiency, ZP removal may reduce potential sources of *Y-DNA* contamination, enabling investigation of male-bias artifacts in PCR-based sex-identification.

PCR amplification of Y-chromosome-specific sequences from ZP-washing supernatants has demonstrated that sperm adhering to the ZP carry amplifiable paternal genetic material, constituting an important source of contamination during embryonic molecular testing [15]. During extended in vitro incubation, protamine disulfide bonds within sperm trapped in ZP gaps gradually reduce, triggering chromatin decondensation. Consequently, sperm genomic DNA becomes more accessible and can be readily released into reaction mixtures, further increasing the risk of false-male bias in PCR-based sex diagnosis [16].

In ruminant IVF systems, relatively high sperm concentrations are routinely used for ovine in vitro fertilization, increasing sperm attachment to the ZP surface and the DNA-contamination risks from trapped sperm [2]. Current human-derived PGT studies have confirmed that zona-associated residual sperm components represent a non-negligible technical risk for PCR-based embryonic genetic testing. Repeated washing cannot completely remove sperm entrapped within the matrix gaps, and ZP removal is regarded as an effective measure to mitigate this type of contamination [17].

Although the literature on human PGT provides mechanistic support, the interfering effect of zona-associated residual sperm components on AMEL-based nested PCR sex typing in ovine embryos has not been systematically validated. Direct experimental evidence remains lacking as to whether ZP removal can eliminate sperm-derived contamination and correct the bias in the PCR-based sex-typing outcomes.

Chen et al. (2025) highlighted that sperm adhering to the ZP during IVF cycles constitute a major source of paternal-originating contamination that directly compromises the diagnostic accuracy of human PGT [18]. This finding indicates that ZP-retained sperm are not mere bystanders but represent a potential systematic contamination source in PCR-dependent embryonic genetic analysis.

Recent studies using ZP-washing fluid, PCR, and embryo-biopsy models have provided evidence of DNA contamination associated with residual sperm. A 2024 PGT investigation using artificial sperm-contamination models suggested that in zona-intact embryos, ZP-attached sperm can be co-harvested with biopsied blastomere cells, producing false-positive paternal-allele signals in PCR outputs. The authors therefore recommended complete ZP stripping prior to biopsy to eliminate such interference [19].

Although *AMEL*-PCR sexing has matured considerably in the field of livestock embryo engineering, systematic reports on directional sex-ratio bias induced by ZP-retained sperm are still lacking. Existing studies mainly focus on primer optimization and amplification system improvement [20], with less attention given to biological contaminants associated with the ZP physical barrier. Notably, embryo sexing and human PGT share strong technical similarities, as both depend on PCR amplification of trace amounts of embryonic DNA and are susceptible to contamination by foreign sperm DNA. Consequently, findings from human embryology studies on ZP stripping to mitigate sperm contamination provide a direct reference for livestock embryo sex-identification workflows [19]. Nevertheless, it remains experimentally unvalidated whether the ZP of early ovine embryos harbors zona-associated residual sperm components, whether such components release Y-chromosomal DNA that interferes with AMEL-PCR interpretation, and whether ZP removal can restore the accuracy of PCR-based embryonic sex typing.

Based on the above research background, we propose the following core hypotheses:

(1) Residual sperm from fertilization may remain on the ZP surface of ovine early-stage blastocysts, and Y-chromosomal DNA carried by these sperm may be released into the PCR system during embryo lysis.
(2) In PCR-based sex determination of embryos with intact zonae pellucidae, residual sperm retained within the ZP may serve as a potential source of Y-chromosomal DNA contamination, contributing to the detection of Y-chromosomal signals and resulting in a biased increase in the proportion of embryos classified as male by PCR genotyping.
(3) Chemical-assisted ZP removal may reduce potential interference from zona-associated residual sperm, thereby bringing PCR-derived sex-determination results closer to theoretical expectations.

## 2. Materials and Methods

### 2.1. Reagents and Instruments

The 50 bp DNA marker was purchased from BaiShan Biotech Co., Ltd., Beijing, China. High-purity low-electroendosmosis agarose and SerRed nucleic acid dye were obtained from Servicebio Technology Co., Ltd., Wuhan, China. A 2× PCR Hero™ Mix was supplied by Foregene Biotechnology Co., Ltd., Chengdu, China. The 2× TransDirect PCR Super Mix kit was purchased from TransGen Biotech Co., Ltd., Beijing, China. Pronase E and acidic Tyrode’s solution (pH 2.5) were obtained from Solarbio Science & Technology Co., Ltd., Beijing, China. DNase I (Cat. No. BS137-10 mg) was purchased from Lanjie Ke Technology Co., Ltd., Beijing, China. Paraformaldehyde and anti-fluorescence quenching mounting medium were purchased from Beyotime Biotechnology Co., Ltd., Shanghai, China. The clean-bench (Model SW-CJ-1B) was obtained from Gene Company Limited, Hong Kong, China. The stereomicroscope (Model SMZ800) was purchased from Nikon Corporation, Tokyo, Japan. The high-speed table-top centrifuge (Model TG16-W) was supplied by Xiangyi Laboratory Instrument Development Co., Ltd., Changsha, China. The PCR instrument (Model ForeAmp-SD696) was obtained from Fanjing Biotechnology Co., Ltd., Chengdu, China. The thermostatic water bath (Model HHS-21-4) was purchased from Jinghong Laboratory Equipment Co., Ltd., Shanghai, China. NanoDrop™ One (Cat. No. 840-317400) was obtained from Thermo Fisher Scientific, Inc., Waltham, MA, USA. The gel imaging system (Model CL 1000) was purchased from Qinxiang Scientific Instrument Co., Ltd., Shanghai, China. The stabilized-voltage electrophoresis power supply (Model DYY-8C) was obtained from Beijing Liuyi Biotechnology Co., Ltd., Beijing, China. The inverted microscope (Nikon ECLIPSE Ti2-U) was purchased from Nikon Corporation, Tokyo, Japan.

### 2.2. Experimental Site and Conditions

The experiment was carried out in Urumqi, Xinjiang, China (43°43′ N, 87°38′ E). Healthy sheep with an average age of 3 years were selected as experimental animals. The sheep were provided with adequate feed, scientific husbandry management, and appropriate light intensity. The animals had ad libitum access to drinking water and received trace-element supplementation. No additional antioxidants were added to the basal diet.

### 2.3. Extraction of Genomic DNA from Blood

Venous blood samples were collected from five healthy non-pregnant ewes and five rams with good body condition. Blood samples were collected into EDTA vacuum blood collection tubes and transported to the laboratory. Genomic DNA from ovine (male and female) blood samples was extracted using the one-step lysis procedure of the TransDirect kit (TransGen Biotech, Beijing, China). Briefly, 1 μL of whole blood was transferred into an EP tube containing 10 μL of PD1 Buffer and incubated at 95 °C for 10 min. After cooling to 12 °C, 10 μL of PD2 Buffer was added for neutralization. The mixture was vortexed thoroughly and used directly as the template for AMEL gene PCR amplification. A 1–2 μL aliquot of the prepared DNA sample was used for nucleic acid concentration measurement (ram: 108.3 ng/μL; ewe: 112.7 ng/μL).

### 2.4. Extraction of Genomic DNA

#### 2.4.1. Embryo Production System

Ovaries were collected from healthy 1-year-old sheep with good body condition at Hualing slaughterhouse in Urumqi, Xinjiang, China. The ovaries were transported to the laboratory within 2 h in normal saline supplemented with 200 IU/mL penicillin–streptomycin at 37 °C. Oocytes were subjected to in vitro maturation for 24 h prior to fertilization.

Oocytes with good morphological quality were selected under a stereomicroscope. To ensure biological replication, three independent in vitro fertilization (IVF) experiments were performed at two-week intervals, using gametes from different donor ewes for each replicate. A total of 200 embryos were obtained, among which 120 embryos were selected for embryonic sex typing. Embryos were divided into two groups: Group A consisted of 50 well-developed Day-6 embryos with intact morphology and intact ZP for analysis of PCR-based sex ratio distribution; Group B consisted of 50 well-developed Day-6 embryos with intact morphology after ZP removal for analysis of PCR-based sex ratio distribution. An additional 20 embryos with intact ZP were selected according to the same criteria for micromanipulation. Partial trophoblast cells were first microaspirated, followed by ZP removal from the embryos. Sex-typing was then performed separately on the micro-sampled trophoblast cells and the corresponding embryo proper. Ram semen was collected using the artificial vagina method. Sperm motility (>70%) and morphological characteristics were evaluated immediately after collection.

#### 2.4.2. Oocyte Maturation

COCs (Cumulus–oocyte complexes) were aspirated from 2 to 8 mm-diameter follicles using 10 mL disposable sterile syringes (Zhiyu Medical, Shanghai, China; YZB/0255-2007) pre-filled with 2 mL of oocyte-aspiration medium.

COCs were classified based on morphological characteristics. Grade A COCs possessed no fewer than three compact layers of cumulus cells, with no signs of degeneration or expansion. Grade B COCs were characterized by fewer than three cumulus-cell layers and oocytes with homogeneous cytoplasm. Grade C corresponded to oocytes lacking cumulus-cell investment, which might display heterogeneous granular cytoplasm. Grade D oocytes exhibited prominent degenerative features, including complete cytoplasmic degeneration or total loss of cumulus cells. Only Grade A and Grade B COCs were used for in vitro maturation. The collected COCs were sequentially washed with oocyte-aspiration medium and maturation medium, then allocated into 4-well plates at a density of 50–70 complexes per well, with 600 μL IVM medium in each well. The COCs were incubated for 22–24 h in a CO_2_ incubator set at 38.6 °C, 5% CO_2_ and 100% relative humidity.

#### 2.4.3. Sperm Preparation

Frozen-thawed semen straws were rapidly retrieved from a liquid-nitrogen container (−196 °C) and immediately immersed in a thermostatically controlled water bath at 37 °C with gentle agitation. Thawing lasted for 2 ± 0.5 min until almost all ice crystals within the straw disappeared. The outer surface of each straw was quickly dried with sterile gauze. The straw was cut at the cotton-plug end, and semen was allowed to flow into a 1.5 mL microcentrifuge tube. Using a 200 μL pipette, the semen was slowly layered beneath 800 μL pre-equilibrated swim-up medium at 38.5 °C. The tube was incubated for 30 min in a CO_2_ incubator maintained at 38.6 °C, 5% CO_2_ and 100% relative humidity. Subsequently, 400–500 μL of the upper-phase medium containing highly motile spermatozoa was collected for subsequent in vitro fertilization [21].

#### 2.4.4. Embryo Culture

Following in vitro maturation, the oocytes were washed three times with IVF medium and transferred into droplets containing 0.1% hyaluronidase. Large expanded cumulus-cell masses surrounding the oocytes were removed through gentle pipetting. Thereafter, oocytes were transferred into wells containing 500 μL of IVF medium. Thawed semen was capacitated in IVF swim-up medium and co-incubated with mature oocytes for fertilization under the same culture conditions as those used for oocyte in vitro maturation. After 22–24 h of incubation, fertilized oocytes were transferred to be equilibrated in in vitro culture (IVC) medium for subsequent embryonic development.

#### 2.4.5. Trophectoderm Microaspiration and Zona Pellucida Treatment

Upon reaching the blastocyst stage, embryos from Group A were incubated in DNase I working solution (1 mg DNase I dissolved in 200 μL nuclease-free water) at 37 °C for 15 min. Immediately after incubation, embryos were recovered and rinsed three times in pre-warmed PBE buffer (PBS supplemented with 1% BSA) to thoroughly remove residual DNase I enzyme and degraded DNA fragments. The washing droplets were examined under a stereomicroscope to confirm clarity and absence of residual sperm. Subsequently, the ZP was softened with 0.25% Pronase-E, and embryos were washed in PBE droplets after each softening step. Individual embryos were then transferred into droplets of acidic Tyrode’s solution. Once dissolution of the ZP was observed, embryos were quickly transferred to PBE droplets for gentle pipetting and subsequently placed into 8-strip PCR tubes for sex-typing. Each embryo was processed individually as described above, and all manipulations were performed on a heated stereomicroscope stage. The detached zonae pellucidae were collected separately in EP tubes containing sterile PBS, subjected to vortex washing, and stored for subsequent lysis and DNA extraction.

Embryos in Group B retained intact zona pellucidae and underwent the same washing procedure as Group A. At least five embryos were subjected to vortexing, and the resulting washing supernatant was collected for DAPI staining.

In addition, 20 DNase I-treated zona-free blastocysts were subjected to micromanipulation. Using a holding pipette and aspiration pipette, 3–5 trophoblast cells were harvested and transferred into a 0.2 mL PCR tube. The remaining embryo proper was transferred into a separate 0.2 mL PCR tube. Each embryo and its corresponding sample were assigned a unique identifier for pairwise matching.

#### 2.4.6. Extraction of Embryonic Genomic DNA

Genomic DNA was obtained from ovine embryonic and trophectoderm cells using the one-step lysis procedure of the TransDirect Kit. Under a stereomicroscope, a single embryo or trophectoderm-cell sample was transferred into 2.5 μL of PD1 lysis buffer, followed by incubation at 95 °C for 10 min. After cooling, 2.5 μL of PD2 neutralization buffer was added, resulting in a final lysis volume of 5 μL per embryo. An aliquot of 1–2 μL was used for DNA quantification. The DNA concentrations were 128.53 ± 1.16 pg/µL for male samples and 131 ± 1.47 pg/µL for female samples, with A_260_/A_280_ ratios ranging from 1.75 to 1.88. The resulting lysis products were used as templates for subsequent PCR amplification.

### 2.5. Primer Design and Synthesis

The *AMEL* gene was used as the sex marker, with copies located on both the ovine X and Y chromosomes: *Ovis aries* amelogenin X-linked (*AMELX*, GenBank accession No. XM_012106213.4) and *Ovis aries* amelogenin Y isoform (LOC132659053, GenBank accession No. XM_060408669.1). *Ovis aries* tyrosine 3-monooxygenase/tryptophan 5-monooxygenase activation protein zeta (*YWHAZ*) was selected as the reference gene (GenBank accession No. XM_012183375.4). Nested primers were designed using BLAST (BLAST: Basic Local Alignment Search Tool 2026.06.12) and Primer Premier 5 software (Table 1). All primers were synthesized by Sangon Biotech (Shanghai, China) Co., Ltd. Double-distilled water was added to adjust the primer concentration based on the OD value of each primer tube. The mixture was vortexed to fully dissolve the primers, and subsequently stored at −20 °C.

### 2.6. Blood PCR System and Conditions

Genomic DNA from sheep blood was used to verify the specificity of the nested inner and outer primers and optimize the amplification conditions. Nuclease-free water served as the negative control. Each 25 μL reaction contained 1 μL DNA template, 0.5 μL each of forward and reverse primers (10 μmol/L), 10 μL 2× Hero Mix, and ddH_2_O to 25 μL. The PCR amplification program was as follows: initial denaturation at 95 °C for 3 min, followed by 18 cycles of denaturation at 94 °C for 30 s, touchdown annealing at 60 °C for 30 s, and extension at 72 °C for 1 min. The second amplification comprised 35 cycles of denaturation at 94 °C for 30 s, annealing at 58 °C for 30 s, and extension at 72 °C for 1 min, followed by a final extension at 72 °C for 5 min. Products were held at 4 °C.

### 2.7. Embryo PCR System and Conditions

Genomic DNA from individual sheep embryos was used to verify and optimize PCR conditions. Nuclease-free water served as the negative control. Each 25 μL reaction contained 5 μL DNA template, 0.5 μL each of forward and reverse primers (10 μmol/L), 10 μL 2× Hero Mix, and ddH_2_O to 25 μL. PCR amplification program was as follows: initial denaturation at 95 °C for 3 min, followed by 18 cycles of denaturation at 94 °C for 30 s, touchdown annealing at 60 °C for 30 s, and extension at 72 °C for 1 min. The second amplification comprised 35 cycles of denaturation at 94 °C for 30 s, annealing at 58 °C for 30 s, and extension at 72 °C for 1 min, followed by a final extension at 72 °C for 5 min. Products were held at 4 °C.

### 2.8. Agarose Gel Electrophoresis Analysis

A 3.5 μL aliquot of each PCR product was loaded onto a 2% agarose gel and electrophoresed at 120 V for 25 min. Amplification products were visualized using a gel imaging system. The DNA marker contained bands of 600, 500, 400, 300, 200, 150, 100, and 50 bp. Two amplification bands were observed: the 130 bp Y chromosome band indicated a male embryo, whilst the 175 bp band indicated a female embryo.

Following agarose gel electrophoresis, nested PCR amplification products from the embryos were submitted to Shanghai China Geneworks Co., Ltd., for further analysis.

### 2.9. Data Analysis

Pearson’s chi-square goodness-of-fit test was used to assess the sex ratio, with χ^2^ values and *p*-values calculated accordingly. The binomial exact test was adopted as a conservative verification to minimize potential bias from the chi-square approximation. The significance threshold was set uniformly at α = 0.05 (two-tailed).

Pearson’s chi-square test of independence, Yates’ continuity-corrected chi-square test, and Fisher’s exact test were applied for intergroup comparisons of sex composition. Fisher’s exact test serves as the gold standard for small sample sizes. Cohen’s h was calculated as the effect size for comparing differences between two proportions, with |h| < 0.2, 0.2–0.5, 0.5–0.8, and >0.8 indicating negligible, small, moderate, and large effects, respectively. All statistical analyses were performed using GraphPad Prism 10 software.

## 3. Results

### 3.1. PCR-Based Sex Classification of Ram and Ewe Blood by AMEL Amplification

**Figure 1:**
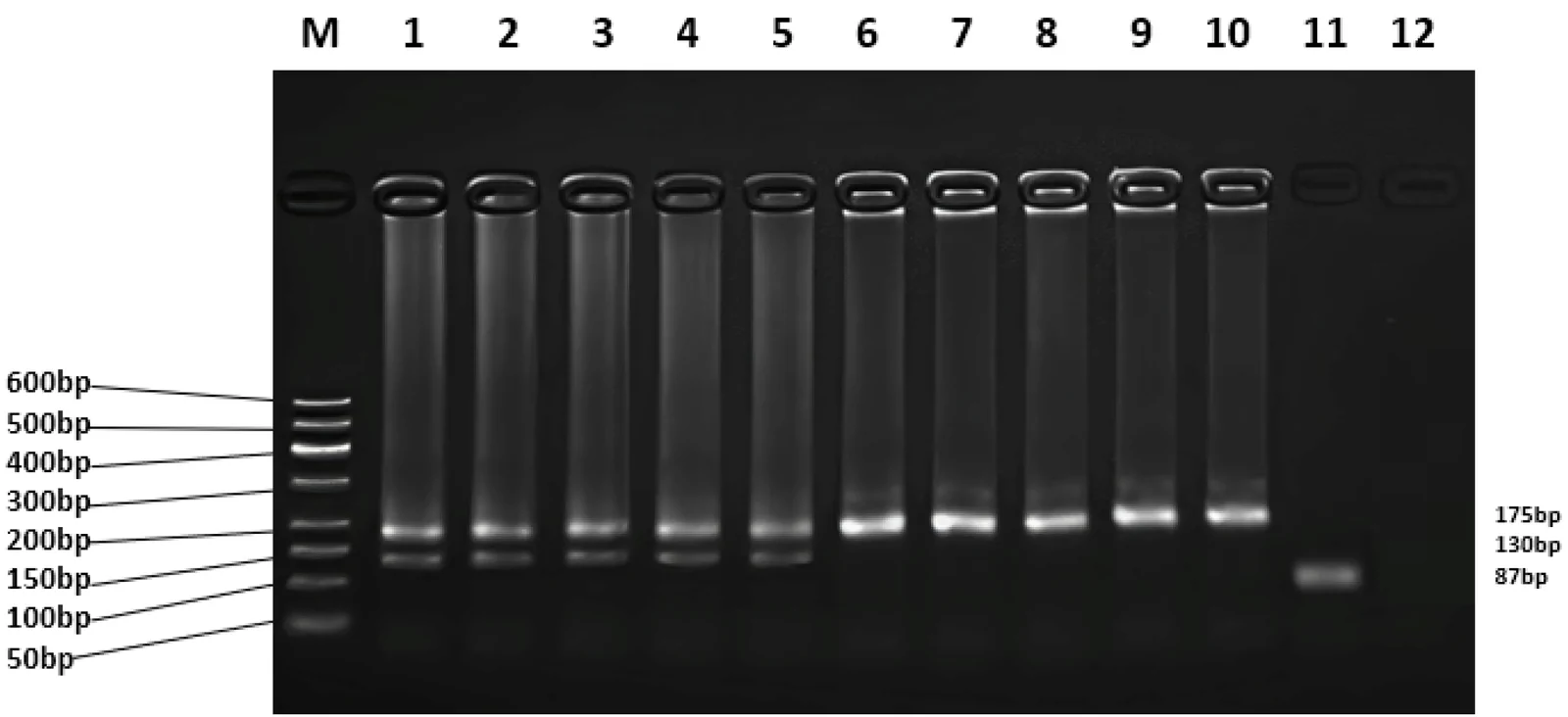
Partial amplification results of *AMELX*, *AMELY*, and *YWHAZ* genes in sheep blood. M: DL600 molecular weight marker. The 130 bp PCR amplicon represents male embryos, while the 175 bp PCR amplicon represents female embryos. Lane 11 shows the amplification product of the *YWHAZ* gene, and lane 12 is the blank control.

We assessed the amplification outcomes of blood samples from male and female sheep via PCR. The *AMELX* and *AMELY* nested primers were validated using sheep blood genomic DNA. After optimizing the annealing temperature, amplification results with good specificity were obtained. Nested PCR generated two specific bands in rams (XY) at approximately 175 bp (*AMELX*) and 130 bp (*AMELY*), respectively (Figure 1), while the internal control produced an 87 bp band. The sizes of both bands were consistent with expectations, and the bands exhibited uniform intensity, indicating good amplification efficiency of the AMEL genes on the X and Y chromosomes. In ewes (XX), which possess only the *AMELX* gene, a single, clear band of approximately 175 bp was detected (Figure 1).

This band was consistent with the expected *AMELX* amplification product, and no *AMELY* (130 bp) band was detected. The internal control gene *YWHAZ* exhibited stable positive amplification in both male and female samples, confirming the validity of the PCR system. No amplification was observed in the ddH_2_O negative control, indicating the absence of detectable contamination.

### 3.2. PCR-Based Sex Classification of Early Ovine Embryos with Intact Zona Pellucida (Group A)

**Figure 2:**
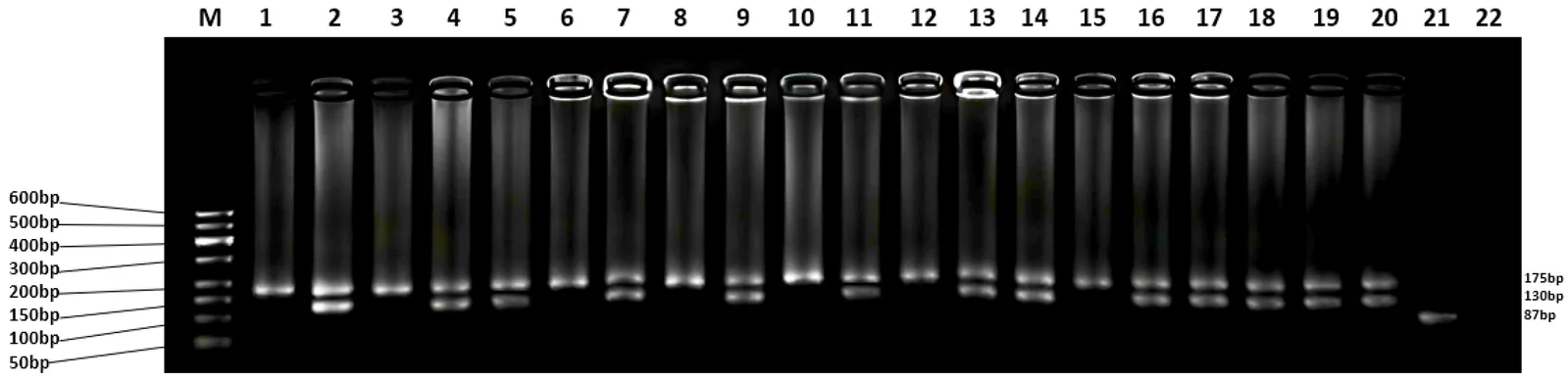
Partial amplification results of *AMELX*, *AMELY*, and *YWHAZ* genes in sheep embryos without zona pellucida removal. M: DL600 molecular weight marker. The 130 bp PCR amplicon represents male embryos, while the 175 bp PCR amplicon represents female embryos. Lane 21 shows the amplification product of the *YWHAZ* gene, and lane 22 is the blank control.

Among the 50 Group A embryos, 17 were classified as female (XX), accounting for 34.0% of the total embryos, and yielded single distinct bands at 175 bp (*AMELX*), consistent with the ewe blood positive control. The reference gene *YWHAZ* was positively amplified in all female embryo samples, confirming the validity of the PCR system (Figure 2). The remaining 33 embryos were classified as male (XY; 66.0%), exhibiting specific bands at 175 bp (*AMELX*) and 130 bp (*AMELY*), consistent with the ram-blood positive control. The reference gene *YWHAZ* showed positive amplification with correct band positions in all male embryo samples, verifying effective PCRs.

The female-to-male ratio was 0.52:1, demonstrating a pattern of elevated male proportion, which suggests a potential source of *Y-DNA* contamination. The chi-square goodness-of-fit test yielded χ^2^ = 5.120 (df = 1, *p* = 0.024), while the binomial exact test produced *p* = 0.033 (significance level α = 0.05). The 95% Wilson confidence interval for the male proportion was 52.2%, −77.6%, excluding the theoretical value of 50%. Cohen’s h was 0.326, indicating a small-to-moderate effect size relative to the theoretical proportion.

This observation suggests that sex genotyping of embryos with intact zonae pellucidae may be affected by certain systematic factors associated with the ZP.

### 3.3. DAPI Staining and Observation of Sperm Residues on the Zona Pellucida of Ovine Early Embryos with Intact Zona Pellucida

**Figure 3:**
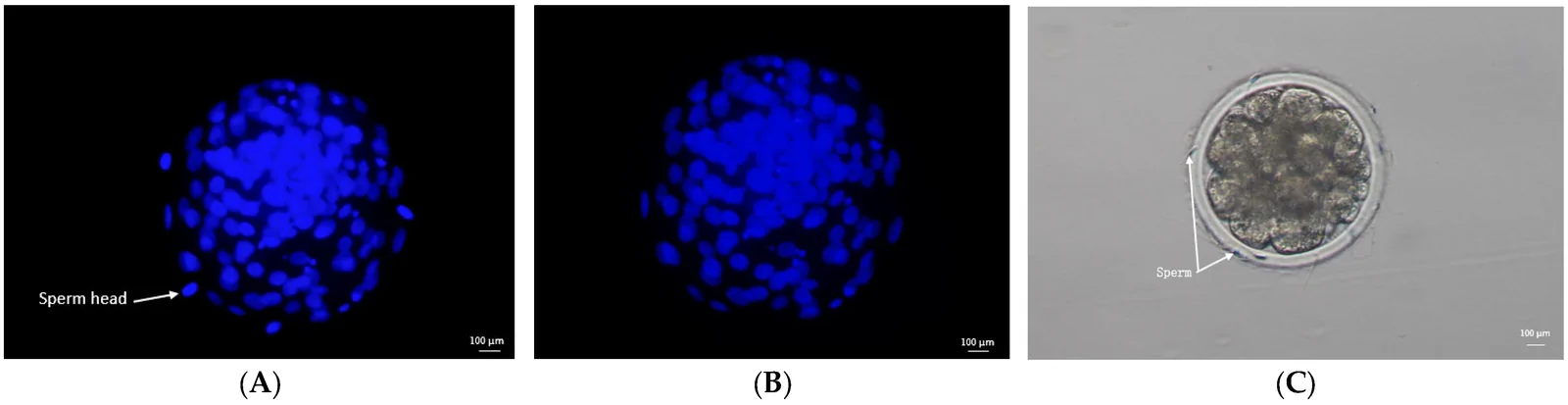
Images of sperm residues on the zona pellucida of ovine early embryos. (**A**) DAPI staining of Day-6 embryos with intact zona pellucida; (**B**) DAPI staining of zona-free embryos; (**C**) bright-field image of embryos with intact zona pellucida.

Figure 3 presents representative micrographs showing sperm residues on the zona pellucida of ovine early-stage embryos. In Panel A, distinct sperm residues exhibiting blue fluorescent sperm heads were observed on the zona pellucida surface or in the perivitelline space. Panel B depicts an embryo with an intact zona pellucida, in which sperm mostly adhered to the outer zona pellucida matrix, while some were embedded at the interface between the zona pellucida and perivitelline space; sperm-tail structures were also visible in some cases. Following zona pellucida removal (Panel C), no clear sperm-originated fluorescent signals were detected around the embryo. These findings suggest that Day-6 early embryos with intact zona pellucida may retain zona-associated residual sperm components or structures containing sperm DNA during in vitro culture and washing procedures.

### 3.4. PCR-Based Sex Classification of Ovine Embryos After Zona Pellucida Removal (Group B)

**Figure 4:**
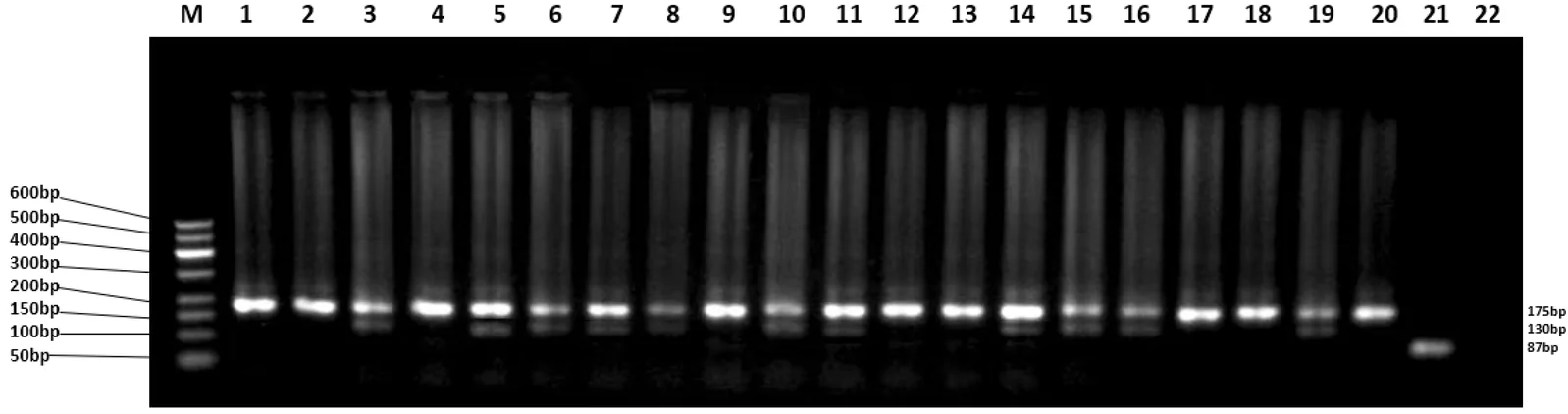
Partial amplification results of *AMELX*, *AMELY*, and *YWHAZ* genes in zona pellucida-deprived sheep embryos. M: DL600 molecular weight marker. The 130 bp PCR amplicon represents male embryos, while the 175 bp PCR amplicon represents female embryos. Lane 21 shows the amplification product of the *YWHAZ* gene, and lane 22 is the blank control.

Among the 50 Group B embryos, 23 were classified as female embryos (XX; 46.0%), showing a single distinct band of 175 bp (*AMELX*), with no detectable 130 bp *AMELY* band (Figure 4). The remaining 27 were classified as male embryos (XY; 54.0%), showing two specific bands at 175 bp (*AMELX*) and 130 bp (*AMELY*) (Figure 4). The reference gene *YWHAZ* was positively amplified in all samples, and no amplification was detected in blank controls.

The female-to-male ratio was 0.85:1, with no statistically significant difference compared with the theoretical 1:1 sex ratio. The chi-square goodness-of-fit test yielded χ^2^ = 0.320 (df = 1, *p* = 0.572), while the exact binomial test gave *p* = 0.672 (significance threshold α = 0.05). The 95% Wilson confidence interval for the male proportion was 40.4– 67.0%, encompassing the theoretical value of 50%. Cohen’s h was 0.090, indicating a negligible effect size.

The absence of a significant male-ratio bias following zona pellucida removal is consistent with our hypothesis regarding zona-associated residual sperm components and indicates that ZP removal may reduce sex-typing bias. However, because ZP removal represents an experimental intervention, its underlying effects cannot be fully established from the present results alone.

### 3.5. PCR-Based Sex Classification of Sheep Embryo Blastomeres by Microaspiration

**Figure 5:**
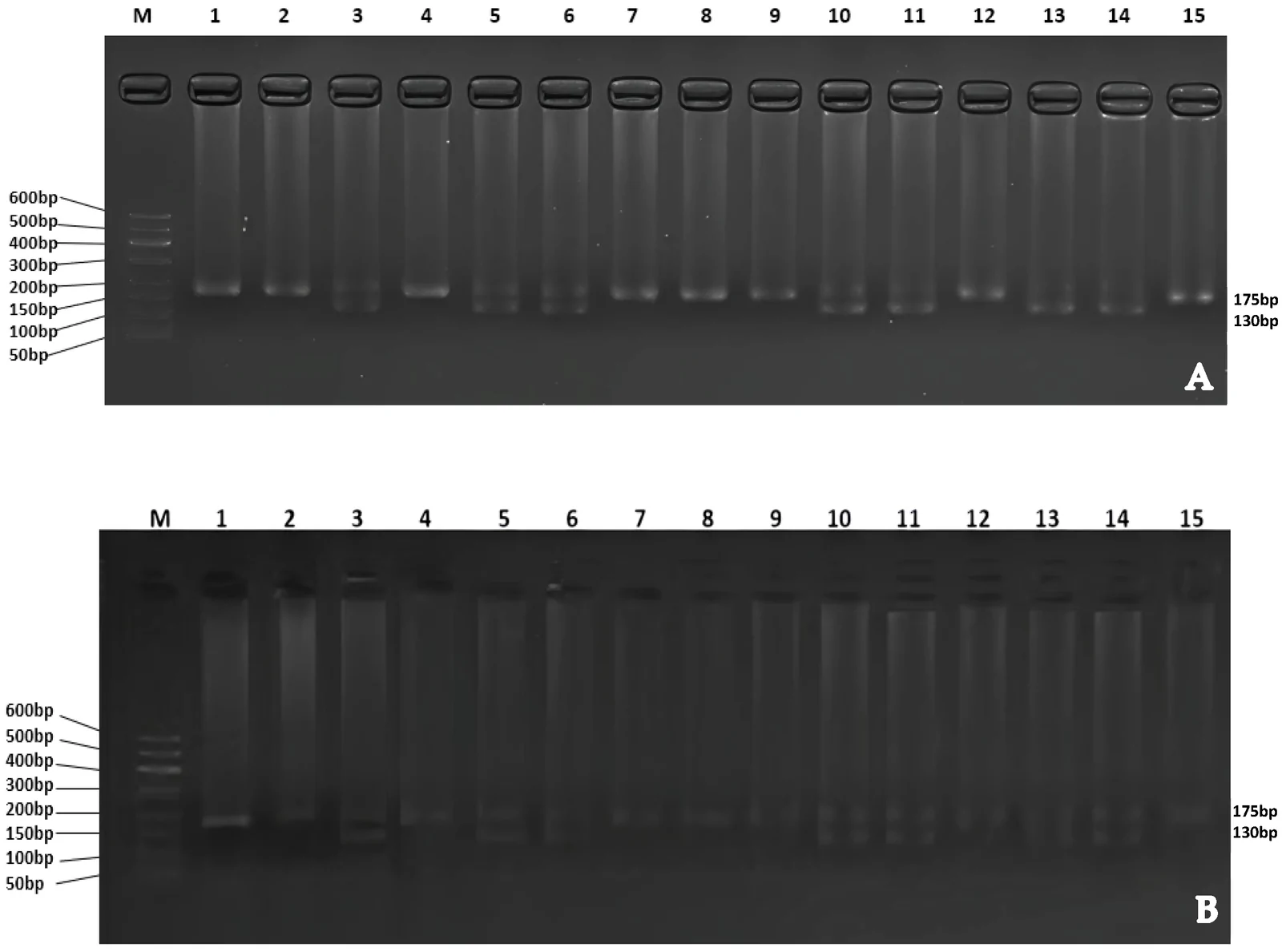
Sex identification results by AMEL-Gene amplification for early-stage sheep embryos with intact zona pellucida versus microaspirated blastomeres. M: DL600 molecular weight marker. (**A**) Identification results of zona-free embryos; (**B**) Identification results of embryonic blastomeres.

Each early-stage zona-free sheep embryo was assigned a unique number and paired with its corresponding microaspirated blastomeres. Following nested-PCR amplification, specific bands were observed at approximately 175 bp (*AMELX*) and 130 bp (*AMELY*). Zona-free embryos showed clear and distinct bands (Figure 5A). Figure 5B shows the sex-identification results of blastomeres. Due to the minute quantity of blastomere samples, the bands in Figure 5B were faint and blurred but remained comparable to those obtained from the corresponding embryos in Figure 5A. Upon comparison, the band size and position of each lane in Figure 5A were largely consistent with those in Figure 5B, indicating that zona pellucida removal may reduce sex-typing bias.

### 3.6. Results of DAPI Staining and AMEL Gene Amplification for Zona Washing Fluids

**Figure 6:**
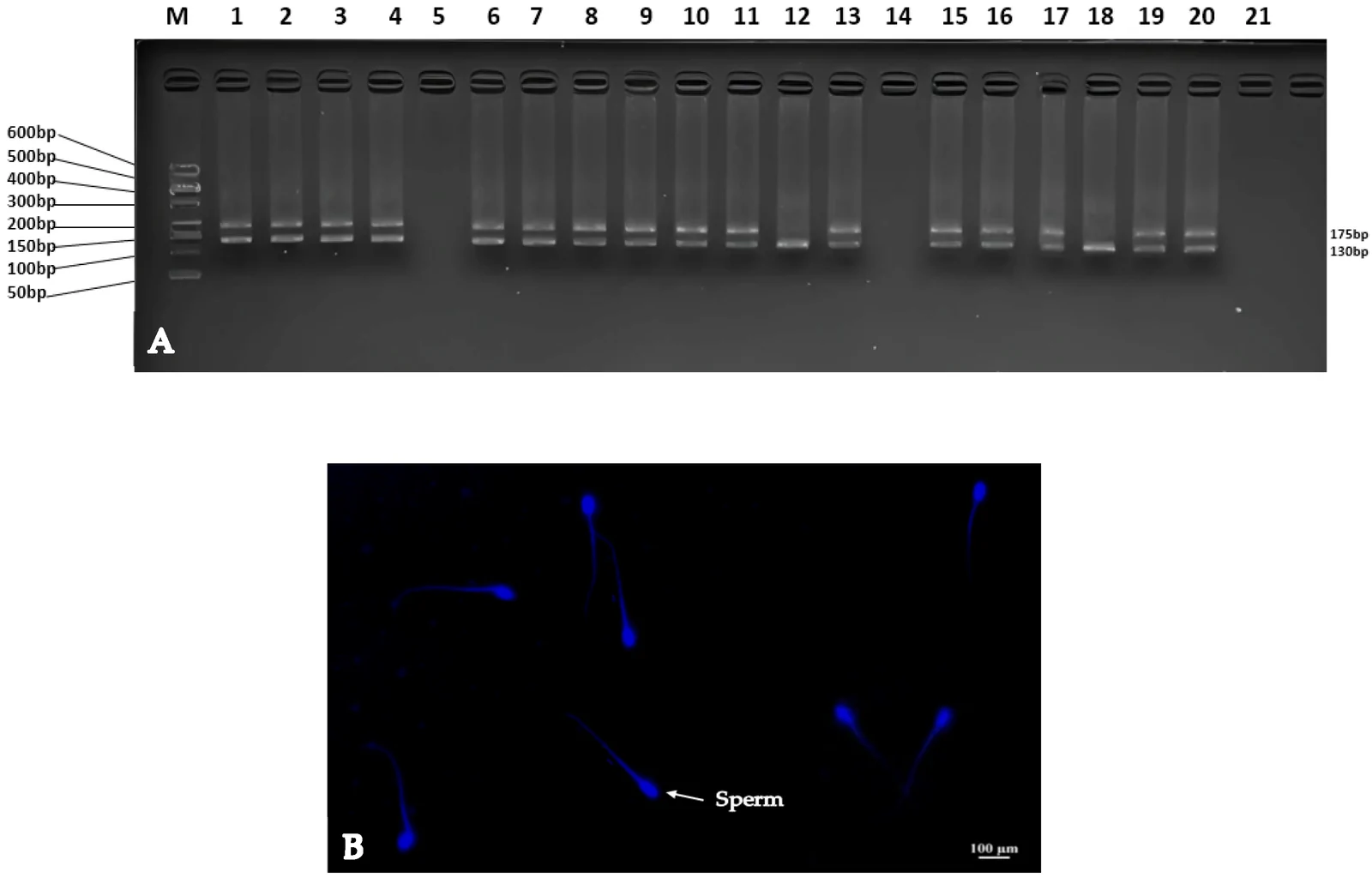
Partial amplification results of *AMELX*, *AMELY*, and *YWHAZ* genes in zona pellucida-deprived sheep embryos. M: DL600 molecular weight marker. (**A**) Electrophoresis results of AMEL gene amplification of zona pellucida washing fluid; (**B**) DAPI staining image of residual sperm in zona pellucida washing fluid. The 130 bp PCR amplicon represents male embryos, while the 175 bp PCR amplicon represents female embryos. Lane 21 is the blank control.

The amplification results of isolated zonae pellucidae collected from Group B were assessed by PCR. As shown in Figure 6A, a 130 bp *AMELY* amplicon was detected in 16 samples (80%), while some samples also yielded the 175 bp *AMELX* band. No amplification was detected in negative controls consisting of PBS washing fluid without embryo exposure. Figure 6B shows DAPI-staining images of washing fluid from Group A embryos with intact zona pellucida, in which structures morphologically similar to sperm were observed. These findings suggest that zona-associated residual sperm components may contain amplifiable Y-chromosomal DNA, and *Y-DNA* released from these sperm may be associated with Y-chromosome signals detected in female embryos during PCR amplification. Nevertheless, the presence of *Y-DNA* in zona pellucida washing fluid does not by itself demonstrate that it directly caused male-bias misclassification of individual embryos.

PCR detection of zona-washing fluids demonstrates contamination at the population level rather than paired validation at the individual-embryo level. The *Y-DNA* detected in washing fluids may originate from free sperm fragments on the zona pellucida surface. However, whether these *Y-DNA* molecules contribute to PCR amplification of lysates from specific embryos cannot be determined from the present experiment. Establishing a direct link between zona-derived *Y-DNA* and Y-signals within individual embryo samples would therefore require paired validation at the single-embryo level.

### 3.7. Sequencing and Alignment Results of PCR Products from Sheep Embryos

**Figure 7:**
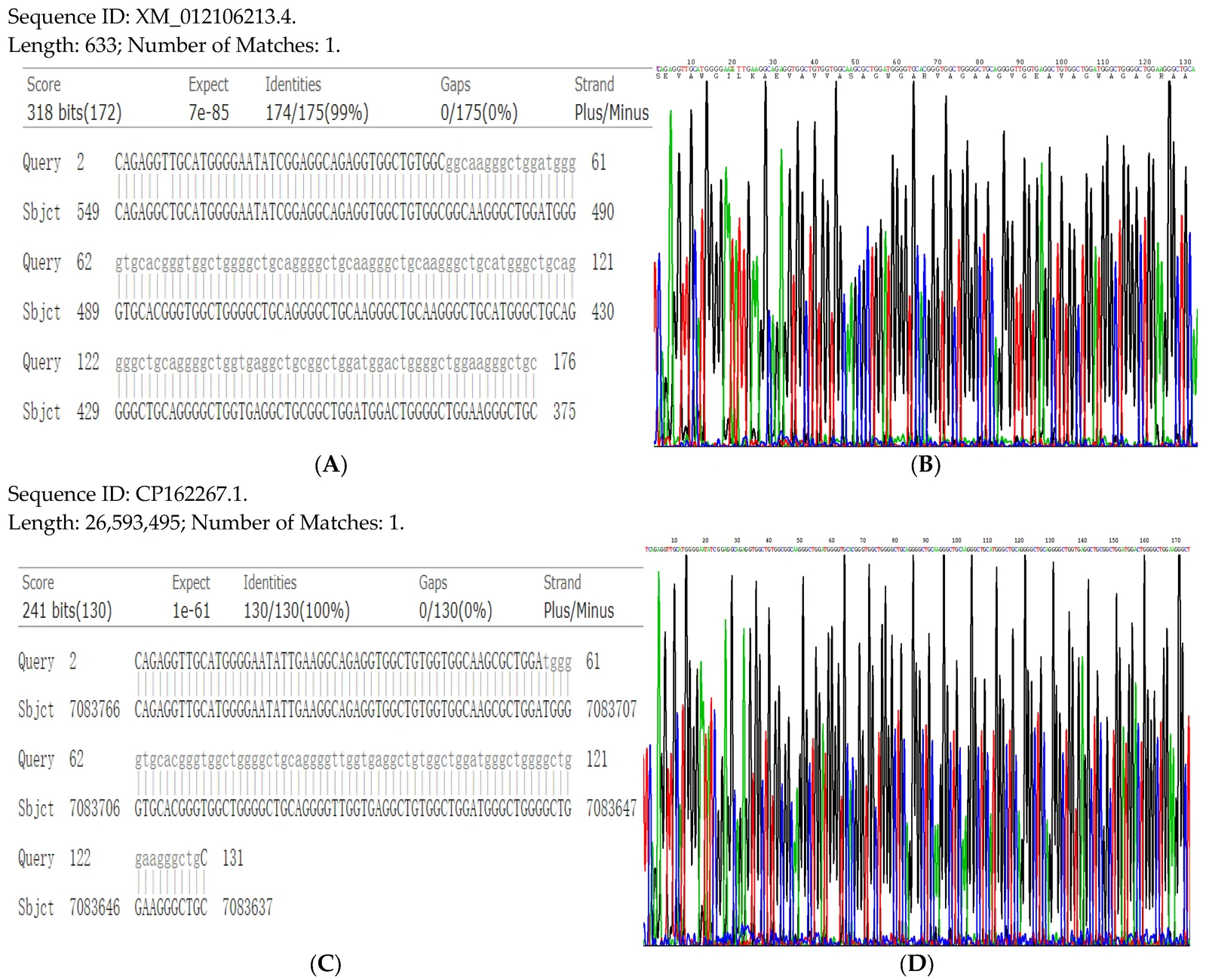
Sequencing results of *AMELX* and *AMELY* gene amplification products from ovine embryos. (**A**): Sequencing results of *AMELX* amplification products. (**B**): Sequencing chromatogram of *AMELX* amplification products. (**C**): Sequencing results of *AMELY* amplification products. (**D**): Chromatogram of *AMELY* amplification products.

Three PCR products corresponding to *AMELX* and *AMELY* were selected for sequencing. The male-associated product was 130 bp in length, while the female-associated product was 175 bp. Both sequences showed 99% identity with the corresponding reference *AMELX* and *AMELY* gene sequences (Figure 7). However, sequencing alone does not independently confirm the biological sex of individual embryos or the origin of the amplified DNA.

### 3.8. Comparison of Sex-Identification Results by AMEL-Gene Amplification Between Zona-Intact and Zona-Free Sheep Embryos

Chi-square analysis showed no statistically significant difference in the male proportion between Group A and Group B (χ^2^ = 1.11, *p* > 0.05). Furthermore, due to the limited sample size (*n* = 50 per group), the statistical power for intergroup comparison was only 23.3%, which was lower than the conventional significance threshold (Table 2). Accordingly, the observed numerical differences should not be considered conclusive evidence of true differences between the two groups. Combined with the DAPI staining results (80% zona-associated residual sperm) and positive AMEL amplification in zona-washing fluids, the present study provides both morphological and molecular evidence that zona-associated residual sperm may serve as a potential source of exogenous *Y-DNA* contamination, thereby explaining the biased results of PCR-based embryonic sex classification. After zona removal, the male proportion in Group B (54. 0%) was closer to the theoretical ratio of 50. 0% (*p* = 0.672), with no obvious sex bias observed (Table 2). The contrasting patterns between the two groups (male-ratio deviation in Group A versus a more balanced distribution in Group B) support the hypothesis that zona-associated residual sperm constitute a major interfering factor in PCR-based sex genotyping. However, the limited sample size, low statistical power, and exploratory nature of the study warrant further investigation. Future studies should use larger sample sizes of no less than 260 embryos per group to achieve 80–90% statistical power and further validate the improvement in sexing accuracy following zona pellucida removal.

## 4. Discussion

In the present study, species-specific primers targeting length polymorphisms between the ovine AMEL genes located on the sex-determining regions of the X and Y chromosomes were designed, and a nested-PCR system for sex identification was optimized. In blood-derived genomic DNA, females produced a single 175 bp *AMELX* amplicon, whereas males produced two distinct bands of 175 bp *AMELX* and 130 bp *AMELY*, consistent with the known phenotypic sex of the animals.

These findings are consistent with previous mammalian sex-identification studies employing the *AMEL* gene. Mishra et al. (2020) suggested that X/Y sequence polymorphisms in the ovine *AMEL* gene are stable and reliable, with nested PCR enabling detection at the single-cell level [2]. De Coster et al. (2022) also used the *AMEL* gene as a molecular marker for accurate sex assignments of porcine early-stage embryos [9]. As a sex-typing marker, the *AMEL* gene exhibits several advantages: it is located within the non-homologous regions of sex chromosomes with one copy on both X and Y chromosomes, thus avoiding gene-dosage interference. Additionally, its high sequence conservation enables versatile primer design across species [10]. However, existing studies on embryonic sex identification have largely focused on assay performance and have not specifically addressed potential interference from exogenous *Y-DNA* contamination derived from zona-associated residual sperm components. In this study, the *YWHA* gene was used as an internal reference gene and produced stable single-band amplification across all samples. This validated the feasibility of our PCR platform and the integrity of template DNA. The use of reference genes can effectively eliminate the interference of PCR inhibitors and avoid false-negative results [22]. In the present study, the amplification results of blood DNA from male and female sheep showed a 99% sequence identity with the corresponding *AMELX* and *AMELY* reference sequences, establishing a reliable technical basis for subsequent PCR-based embryo sex classification. However, this study lacked a gold-standard method for validating embryo sex typing. Although AMEL-based nested PCR has been widely applied for embryo sex determination of cattle [6], pigs [23], and sheep [2], previous studies primarily established the analytical performance of the assay and cannot directly confirm the accuracy of PCR-based sex classification in individual intact embryos in the present study.

PCR-based sex-typing outcomes were evaluated at the analytical level. A significant male-ratio bias (66.0%) was observed in Group A, whereas the male proportion in Group B, following zona pellucida removal, was 54.0%, closer to the expected 1:1 distribution. In addition, nested PCR-based sex typing of microaspirated blastomeres from zona-free early ovine embryos was consistent with the corresponding zona-free embryos. This suggests that zona pellucida removal may reduce sex-typing bias and has the potential to reduce or eliminate zona-associated sperm-derived interference, represented by the occurrence of Y-chromosome signals in female embryonic samples. This phenotype is consistent with the biological hypothesis of possible exogenous *Y-DNA* release from zona-associated residual sperm components.

The novelty of this study does not lie in the AMEL-based PCR, nested PCR methodology, or the sex identification of zona-free ovine embryos, but in investigating zona-associated residual sperm as a potential source of exogenous *Y-DNA* interference and elucidating the resulting sex-typing bias in PCR-based embryonic sex identification. The study therefore provides analytical evidence of a potential source of sex-typing interference rather than formal diagnostic validation, as lambing outcomes were not used as a gold standard. Future studies should use larger sample sizes (a minimum of 260 samples per group is recommended to detect a 12% difference with 80% statistical power) and perform rigorous diagnostic validation based on lambing phenotypes to further confirm the correlation between zona-associated residual sperm contamination and sex-typing bias in ovine embryos.

Only one sperm successfully penetrates the zona pellucida to achieve fertilization, and excess sperm can bind to ZP3 glycoprotein receptors and remain attached to the zona pellucida outer surface or become embedded within matrix gaps [24]. Chang et al. (2026) reported that even non-penetrating sperm can establish tight acrosomal binding to ZP3, generating physically persistent sperm residues that cannot be easily eliminated [25].

Given the limited sample size (*n* = 50 per group), the statistical power for direct intergroup comparison was only 23.3%, which was lower than the conventional statistical significance threshold. Therefore, the observed numerical differences should not be interpreted as conclusive evidence of true biological differences between the two groups. Nevertheless, the significant male-ratio bias in Group A, the more balanced distribution in Group B, and the supporting molecular evidence are consistent with the hypothesis that zona-associated residual sperm components serve as a source of exogenous *Y-DNA* contamination. However, the limited sample size, low statistical power, and exploratory nature of the present study warrant further investigation. Therefore, future studies should use a larger sample size of no less than 260 embryos per group to verify whether ZP removal improves the accuracy of PCR-based sex classification with adequate (80–90%) statistical power.

As a physical barrier during fertilization, the zona pellucida may retain residual sperm on its surface, which can act as a potential source of exogenous *Y-DNA* contamination and result in male genotyping bias. In the present study, DAPI fluorescence staining indicated residual sperm in 80.0% of zona-intact embryos, a proportion consistent with physiological polyspermy-related phenomena during ovine in vitro fertilization. Additionally, the *AMELY* fragment (130 bp) was amplified from 80.0% of embryo-zona-washing supernatants, confirming that these zona-associated sperm residues can contain amplifiable Y-chromosomal DNA. Wen et al. (2024) similarly reported abundant sperm adhesion to the zona pellucida of conventional-IVF embryos, representing a major source of paternal DNA contamination during embryonic genetic testing [26]. It was found that prolonged in vitro incubation of capacitated ovine sperm increases sperm decondensation [27]. In vitro culture perturbs protamine cross-linking stability, relaxing nuclear architecture and promoting DNA release, which may distort embryonic PCR-based readouts [27].

We hypothesize that during embryo lysis, sperm trapped within the zona pellucida may undergo lysis alongside the oocyte. Highly compact protamine-DNA complexes within these sperm are disintegrated by DTT and proteinase K, releasing Y-chromosome DNA that co-serves as a PCR template together with intrinsic embryonic genomic DNA. This process could misclassify genetically female (XX) embryos as male (XY).

DAPI staining showed that residual sperm were predominantly localized to the outer zona pellucida layer and perivitelline space, with some sperm heads embedded within the zona matrix and tails remaining free or fragmented. Such morphological distribution closely matches the sperm-residue patterns documented by De Coster et al. (2022) [9]. Owing to the porous glycoprotein architecture of the zona pellucida, the attached sperm cannot be completely removed by standard embryo-washing procedures. Sperm-chromatin status governs whether residual sperm interfere with PCR amplification. Mature sperm chromatin is tightly condensed through protamine-mediated disulfide-bond cross-linking, limiting DNA accessibility during lysis [27]. However, the successful amplification of Y-chromosome sequences from zona-washing fractions in the present study implies that partially trapped sperm undergo chromatin decondensation triggered by redox shifts or proteolytic activity in the in vitro culture, releasing amplifiable DNA templates. Ribas-Maynou et al. (2021) corroborated that protamine disulfide bonds gradually become reduced in in vitro sperm incubation, increasing DNA accessibility [16].

Zona pellucida removal is widely applied in assisted reproduction. Fan (2022) employed acidic Tyrode’s solution for zona pellucida removal in mouse embryos in an embryo transfer study [11]. However, acidic Tyrode’s treatment may affect embryo developmental competence and gene expression. Previous studies have reported effects on both pre- and post-implantation development following zona pellucida removal [28].

Pronase-E is also used to soften or remove the zona pellucida, especially in embryo micromanipulation [29]. However, residual protease following treatment may interfere with subsequent molecular procedures. Although repeated washing steps are routinely performed to eliminate enzyme solution during embryo handling, incomplete removal may affect downstream lysis or DNA recovery. Studies on primary chondrocyte culture reported that Pronase-E pretreatment combined with collagenase digestion could significantly improve cell yield. Meanwhile, enzymatic treatment can also modify cell-membrane surface proteins and extracellular matrix, which may compromise DNA recovery efficiency from treated samples or introduce PCR inhibitors, interfering with subsequent molecular detection [29].

Treatment with acidic Tyrode’s solution or Pronase-E may release cellular debris from the embryo surface or alter cell-membrane permeability, resulting in increased fragmentation of DNA released into the surrounding environment. Fragmented DNA may reduce amplification efficiency during whole-genome amplification (WGA) and PCR assays, which may induce allelic amplification bias and further impair the accuracy of sex-typing [30].

At the PCR-based analytical level, zona pellucida removal reduced sex-typing bias. ZP1-ZP4 glycoproteins constitute over 90% of the zona pellucida dry weight, and upon embryo lysis, these glycoproteins may leak into reaction mixtures and inhibit Taq DNA polymerase activity [31]. Compared with Group A, amplicons derived from Group B exhibited sharper band profiles and lower background noise, which may be attributable to the removal of zona pellucida glycoprotein inhibitors. Sándorová et al. (2026) similarly reported that lysates from zona-intact embryos contain abundant protein-based PCR inhibitors that suppress Taq-polymerase performance and reduce amplification efficiency [22].

Nevertheless, zona pellucida removal is not without potential risks. Acidic treatment may damage embryonic cell membranes, and prolonged exposure can trigger cell dissociation or embryo death [32]. In the present study, treatment duration was limited to ≤60 s, and no obvious adverse effects on embryo survival or development were detected. For clinical scenarios requiring pre-transfer embryo sex diagnosis, however, the potential impact of zona pellucida removal on implantation capacity must be carefully considered against diagnostic benefits [11].

Previous livestock-sexing studies using bovine [6], porcine [24], and ovine embryos generally reported sex ratios close to 1:1 for zona-intact embryos [2]. To our knowledge, the present study is the first to report a male-ratio bias in zona-intact ovine embryos and experimentally link this bias to sperm residues trapped within the zona pellucida, which are a plausible source of false-male sex assignments. These findings support the possibility that sperm retained by the zona pellucida may contribute to exogenous *Y-DNA* interference and false-male sex assignments in PCR-based embryo sex classification. This finding provides a basis for improving the accuracy and standardizing the operating procedures of ovine embryonic PCR-based sex classification distribution.

Notably, physicochemical properties of the zona pellucida vary across mammalian species. In ruminants such as sheep and cattle, high abundance and stable matrix integration of the ZP3/ZP4 complex enable firmer physical retention of bound sperm [33]. Furthermore, ovine IVF typically requires high sperm-insemination concentrations to achieve adequate fertilization efficiency. This generates dense periovular sperm clouds and increases sperm-retention loads on the zona pellucida surface [34].

## 5. Conclusions

This study established a nested PCR system for sex identification of early ovine embryos based on the *AMEL* gene. Zona-intact embryos showed a male-biased sex distribution, whereas zona-free embryos exhibited sex ratios consistent with the theoretical expectation. These observations are consistent with the biological hypothesis that exogenous Y-chromosomal DNA derived from zona-associated sperm may serve as a potential contamination source in PCR-based embryo sex identification. The results provide a fundamental basis for subsequent comparative analysis of zona-intact and zona-free embryos and warrant further investigation of residual sperm as a critical interfering factor.

Although the reliability of this *AMEL*-based nested PCR system has been validated through blood DNA amplification, embryonic detection, and sequencing verification, as well as supported by previously published ovine embryonic sexing studies, these assessments cannot fully confirm the accuracy of PCR-based sex classification at the individual-embryo level. The novelty of this study, therefore, lies in identifying zona-associated residual sperm as a potential source of exogenous *Y-DNA* interference rather than in the AMEL-based PCR or nested PCR methodology itself. The findings provide analytical evidence of a potential source of sex-typing bias rather than formal diagnostic validation, as lambing outcomes were not used as a gold standard. Future studies should use larger sample sizes (a minimum of 260 samples per group is recommended to detect a 12% difference with 80% statistical power) and perform rigorous diagnostic validation based on lambing phenotypes to further validate the correlation between zona-associated residual sperm contamination and sex-typing bias in ovine embryos.

## Figures and Tables

**Table 1:** Table of nested primer sequences.

Gene	Primer Sequences (5′–3′)	Annealing Temperature (°C)	Product Length (bp)
*AMELX*-F1	CATGGTGCCAGCTCAGCAG	60 °C	175
*AMELX*-R1	CCGCTTGGTCTTGTCTGTTGC		
*AMELX*-F2	GCAGCCCTTCCAGCCCCAG	58 °C	130
*AMELX*-R2	CAGAGGTTGCATGGGGAATAT		
*YWHAZ*-F1	AGGAGCCCGTAGGTCATCTT	58 °C	87
*YWHAZ*-R1	TCTCGAGCCATCTGCTGTTT		

**Table 2:** Comparison of PCR-based sex classification of sheep embryos in Group A and Group B by AMEL gene amplification.

Group	Female-to-Male Ratio	Male Proportion
Group A (with intact zona pellucida)	0.52:1	66.0%
Group B (with zonae pellucidae removed)	0.85:1	54.0%
Theoretical expectation	1.00:1	50.0%

## Data Availability

The original contributions presented in the study are included in the article; further inquiries can be directed to the corresponding authors.

## Abbreviations

The following abbreviations are used in this manuscript:

PBE	PBS + 1%BSA
IVM	In vitro maturation medium
IVF	In vitro fertilization medium
IVC	In vitro culture medium
COCs	Cumulus–oocyte complexes
DNA	Deoxyribonucleic acid
PBS	Phosphate-buffered saline
mL	Milliliter
µL	Microliter
h	Hour (s)
min	minute (s)
s	Second (s)
CO_2_	Carbon dioxide
PCR	Polymerase chain reaction
AMEL	Amelogenin
*YWHAZ*	Tyrosine 3-monooxygenase-activated protein zeta
ddH_2_O	Double-distilled water
bp	Base pair (s)
mol	Mole
pg	picogram
ng	nanogram
df	Degrees of freedom
